# Dynamic Responses in Brain Networks to Social Feedback: A Dual EEG Acquisition Study in Adolescent Couples

**DOI:** 10.3389/fncom.2017.00046

**Published:** 2017-05-31

**Authors:** Ching-Chang Kuo, Thao Ha, Ashley M. Ebbert, Don M. Tucker, Thomas J. Dishion

**Affiliations:** ^1^Electrical Geodesics Inc.Eugene, OR, United States; ^2^NeuroInformatics Center, University of OregonEugene, OR, United States; ^3^Department of Psychology, Arizona State UniversityTempe, AZ, United States; ^4^T. Denny Sanford School of Social and Family Dynamics, Arizona State UniversityTempe, AZ, United States; ^5^Department of Psychology, REACH Institute, Arizona State UniversityTempe, AZ, United States; ^6^Department of Psychology, University of OregonEugene, OR, United States

**Keywords:** adolescent couples, social interaction, dense-array EEG, principal component analysis, event-related potential, source localization

## Abstract

Adolescence is a sensitive period for the development of romantic relationships. During this period the maturation of frontolimbic networks is particularly important for the capacity to regulate emotional experiences. In previous research, both functional magnetic resonance imaging (fMRI) and dense array electroencephalography (dEEG) measures have suggested that responses in limbic regions are enhanced in adolescents experiencing social rejection. In the present research, we examined social acceptance and rejection from romantic partners as they engaged in a Chatroom Interact Task. Dual 128-channel dEEG systems were used to record neural responses to acceptance and rejection from both adolescent romantic partners and unfamiliar peers (*N* = 75). We employed a two-step temporal principal component analysis (PCA) and spatial independent component analysis (ICA) approach to statistically identify the neural components related to social feedback. Results revealed that the early (288 ms) discrimination between acceptance and rejection reflected by the P3a component was significant for the romantic partner but not the unfamiliar peer. In contrast, the later (364 ms) P3b component discriminated between acceptance and rejection for both partners and peers. The two-step approach (PCA then ICA) was better able than either PCA or ICA alone in separating these components of the brain's electrical activity that reflected both temporal and spatial phases of the brain's processing of social feedback.

## Introduction

Adolescence is a critical time for developing significant peer and romantic relationships (Steinberg and Morris, 2001). Social rejection is a normal developmental challenge, but it may figure importantly in pathological forms of adolescent anxiety and depression (Masten et al., 2012; Moor et al., 2012; Silk et al., 2012). Previous functional magnetic resonance imaging (fMRI) studies found that the dorsal anterior cingulate cortex (dACC) is activated in social rejection (Eisenberger et al., 2003). The ventral anterior cingulate cortex (vACC) and specifically the subgenual region (subACC) appear sensitive to social feedback, particularly acceptance from unfamiliar peers (Somerville et al., 2006, 2010; Guyer et al., 2012; Masten et al., 2012).

In addition to fMRI studies, event-related potential (ERP) studies have addressed the neural processing of social feedback in adolescents. The P3 component of the ERP (typically the third positive wave) is a particularly sensitive measure of the attention and memory processes related to the cognitive processing of significant information (Polich, 2007; Volpe et al., 2007). In the Cyberball laboratory task, subjects experience rejection when it is their turn to be passed the ball but it is passed to someone else. Using this task Crowley et al. showed that frontal, slow wave activity (580–900 ms post-stimulus) among young adults, which was more negative for participants reporting more distress and more positive for participants reporting less distress (Crowley et al., 2009). This finding was replicated in a sample of children between 8–12 years old, where a similar slow wave was found associated with a larger P3 component in response to exclusion (Crowley et al., 2010). Another study (Gutz et al., 2011) using the Cyberball paradigm found that exclusion influenced both a fronto-central P3a and a more parietal P3b component. The P3a was related to later negative mood and the P3b was involved in processing the intensity of the exclusion itself. A similar study identified a larger P3b, indicating enhanced attentional activation to exclusionary events (Themanson et al., 2014). The P3b activation was associated with self-reported social distress following prolonged social exclusion.

An important question is whether neural responses in laboratory tasks with unfamiliar peers can be generalized to the more emotionally significant responses to acceptance and rejection from romantic partners. Adolescents' relative inexperience with romantic relationships, coupled with the high likelihood of breakups, creates a highly vulnerable context (Ha et al., 2012). Not surprisingly, romantic rejection has been related to problem behavior (Furman et al., 2008) and depression (Monroe et al., 1999; Ha et al., 2014). To the best of our knowledge, adolescent romantic relationships have not been examined before, possibly due to practical challenges of recruiting young dating couples and creating believable paradigms that elicit romantic partner acceptance and rejection.

In the present study, we examined dense array EEG measures associated with events of acceptance and rejection between both romantic partners and unfamiliar peers as they engaged in virtual social feedback. Specifically in the Chatroom Interact Task, the subjects indicted whether they preferred a given person for a planned chat on a specific topic, such as movies or sports. Dual 128-channel dEEG systems were synchronized, so that both romantic partners were recorded simultaneously. We investigated the dEEG measures for neural signs of differential attention to acceptance and rejection, specifically related to responses from the partner versus an unfamiliar peer. To improve the separating of overlapping ERP components (such as P3a and P3b), we employed a two-step temporal principal component analysis (PCA) followed by a spatial independent component analysis (ICA) to separate the underlying neural generators of the superimposed scalp voltage patterns (Dien et al., 2005). Compared to standard ERP component analysis that provides local information of amplitude and time courses for brain signals (Cacioppo et al., 2015), the two-step component analysis approach provides comprehensive information about reliable and distinct components that contribute to the dense-array recordings (Foti et al., 2009). More precisely, the two-step component analysis approach provides component time courses and near-dipolar scalp projections, suggesting the component reflects a coherent neural source (Delorme et al., 2012). To explore the possible neural sources with realistic physical modeling, source estimation was conducted for each component with a linear inverse solution (LORETA; Pascual-Marqui et al., 1994) and a high-resolution of electrical head models (Li et al., 2016).

We hypothesized that the frontal and limbic (anterior cingulate, insula) neural responses during social rejection observed in previous laboratory tasks (Crowley et al., 2009, 2010) would generalize to this more naturalistic virtual social feedback paradigm. Furthermore, we hypothesized that feedback from a romantic partner would be more intense and perhaps engage more immediate emotional and limbic activity than feedback from an unfamiliar peer. These hypotheses were tested with the two-step PCA/ICA component analysis, separating the components underlying the cerebral networks of initial attentional engagement (P3a) and more extended cognitive evaluation (P3b), and then statistically analyzing the component responses as a function of acceptance or rejection by the partner or the peer.

## Materials and methods

### Participants

Ninety-nine adolescent couples aged 14 to 18 years participated in the current study (Ha et al., 2016). The majority of these couples were heterosexual (*N* = 91) and 8 were same sex couples (1 male and 7 female couples). Relationship length was diverse with 34.2% of adolescents reporting being in a relationship less than 6 months, 38.4% between 6 and 12 months, and 27.4% for longer than a year. All participants who had fewer than 10 artifact free trials across all conditions were excluded from further analyses. Our final EEG study sample consisted of 75 subjects with normal or corrected-to-normal vision (51 males and 24 females, *M* = 16.44 years, *SD* = 0.83; range 15–18 years). All participants reported no history of neurological disorders nor were they taking medications that are known to affect the EEG (e.g., anticonvulsants). This study was carried out in accordance with the recommendations of The Institutional Review Board of Arizona State University, which approved the study protocol. Parents provided written informed consent and adolescents provided written informed assent prior to participation. Participants each received $40 for the laboratory session.

### Stimuli and experimental design

**Figure 1:**
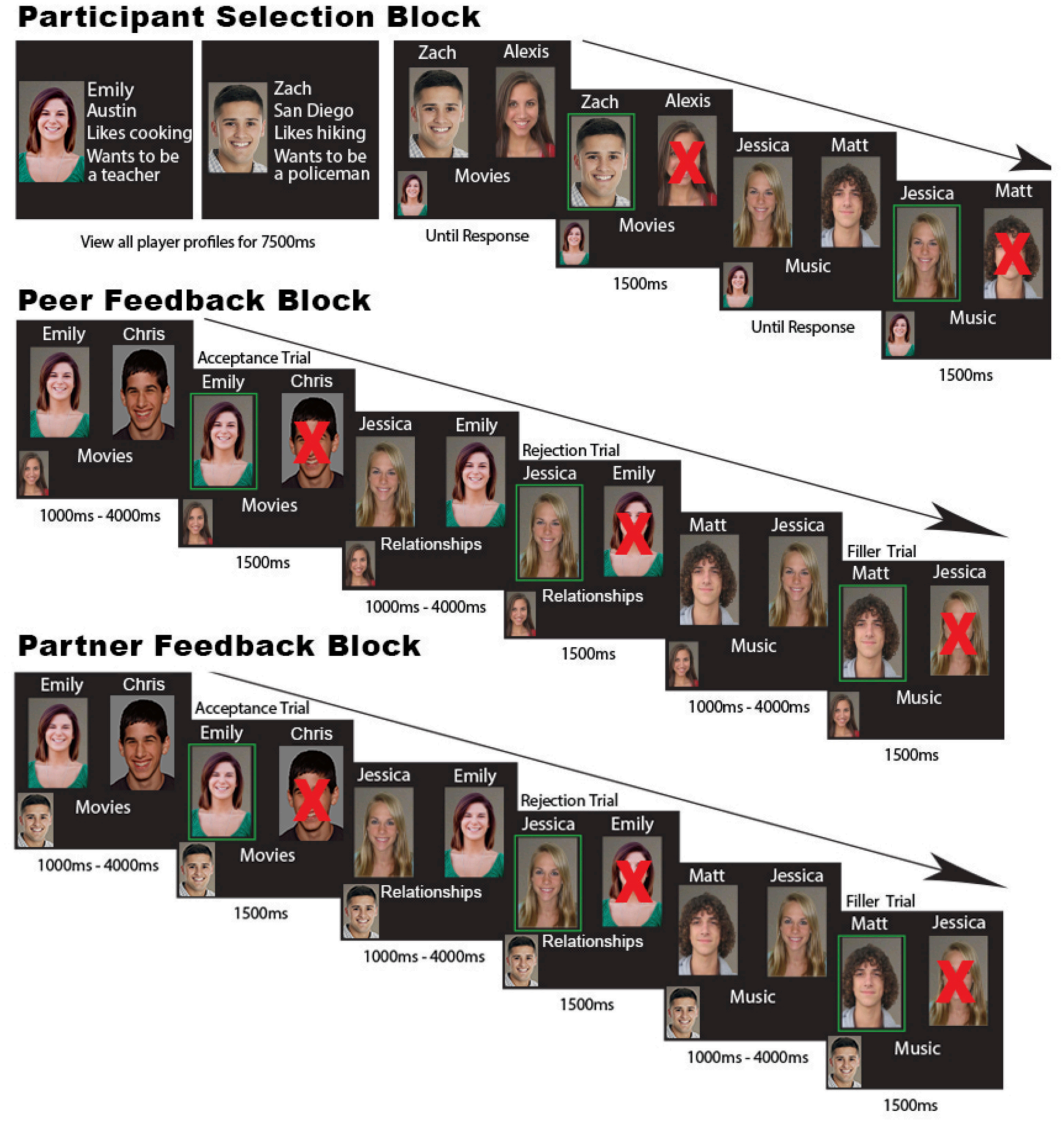
Representation of the Chatroom Interact Task. During the participant SELECTION block, both participants viewed 6 player profiles as an introduction to the task (2 participant profiles; “Emily” and “Zach,” two additional fictive males and females). Then, participants were instructed to choose the person who they would like to talk to regarding the topic displayed under the players' photos. In this particular example, we showed the player timeline of Emily (picture shown in the left corner) who was making selections between 2 players with a “left” or “right” button press. The selected or “accepted” player was subsequently highlighted with a green square on their picture. The unselected or “rejected” player was labeled with a red “X” over their picture. Following the participant selection block, fictitious feedback was provided by peers and the romantic partner in the FEEDBACK blocks, indicating whether the participant was accepted or rejected. Feedback was randomized by the experimenters and believed by the subjects. In the peer FEEDBACK blocks, participants would see their peers making selections. In this particular example, Emily (participant) viewed that Alexis (peer, displayed in the left corner) chose the participant to talk about movies, which was a peer acceptance trial. Then Alexis (peer) chose Jessica (peer) over Emily (participant) to talk about relationships, which was a peer rejection trial. This was followed by a filler trial where the participant, Emily, was not displayed as an option. In the romantic partner FEEDBACK block, participants viewed their romantic partners' selections. In this case, Emily (participant) watched Zach (romantic partner) selecting Emily to talk about movies, which was a romantic partner acceptance trial. However, Zach chose Jessica (peer) over Emily (participant) to talk about relationships, which was a romantic partner rejection trial. Note that only the peer and romantic partner FEEDBACK blocks were analyzed in the current study.

Adolescent couples participated in a Chatroom Interact Task, designed to investigate neural reactions to online virtual peer acceptance and rejection (Guyer et al., 2009; Silk et al., 2012). We adapted this task to fit a dyadic context where both romantic partners participated simultaneously to elicit both rejection and acceptance from a romantic partner and unfamiliar peers (Figure 1). Participants were told a cover story explaining that they would be having online video chats with other adolescent players from three different universities. These video chats would be based on each participant's choices to talk with another participant about a variety of topics (e.g., dating, books, future plans, and parties). In reality, these video chats would never occur and we were merely interested in adolescents' neural responses to social feedback (acceptance versus rejection) from peers and their romantic partner. A personalized chat profile was created, providing information about a player's first name, interests, and proposed future career plans along with their picture.

In the first “selection” block, participants selected their conversation partners. They viewed 4 other player profiles (2 males and 2 females) and their partners' profile, each for 7,500 ms. Participants were instructed to choose the person who they would like to talk to about the topic displayed under the players' photos. Two photos were shown and the participant selected with a “left” or “right” button press that corresponded with the picture. The selected or “accepted” player was subsequently highlighted with a green square around their picture. The unselected or “rejected” player was labeled with a red “X” over their picture for 1,500 ms. The selection block consisted of 45 randomized trials on 15 various topics, each repeating three times in a randomized order. Of the 45 trials, 30 trials included the participant's romantic partner as one of the options to choose from when randomly paired with one of the other 4 players.

Data for the current study were obtained from the “feedback” blocks where participants were able to see the other players' choices including one partner selection round and four peer selection rounds. During the peer feedback blocks, the profile picture of the peer player making selections was displayed in the bottom corner of the screen, along with the topic and the pictures of the 2 players being selected. The time it took for the peer player to select was set to a random iteration between 1,000 and 4,000 ms. Feedback was provided to participants by presenting them with their photo highlighted with either a green square (acceptance) or covered by a red cross (rejection), which lasted 1,500 ms. Overall, the peer feedback blocks contained a randomized order of 45 trials, where 30 trials consisted of the participant randomly paired with one of the four peers and 15 trials including either two randomly paired peers. A similar set up was used for partner feedback blocks. However, the only change from the peer feedback blocks was that the participant would receive feedback about their romantic partner's choices. Thus, participants could be either accepted or rejected by their romantic partner. This procedure was repeated with different players for a second round. The order of the partner and peer feedback blocks was randomized. After the Chatroom Interact Task, participants have fully debriefed that all feedback was computer manipulated.

### EEG acquisition

Continuous EEG was acquired with dual 128-channel HydroCel Geodesic Sensor Net (EGI, Eugene, OR, USA; http://www.egi.com/) using Net Station 4.5 software. EEG electrodes were distributed across the whole head surface with an inter-sensor distance of approximately 3 cm (Tucker, 1993). All electrode impedances were below 70 KΩ before recording was started (Ferree et al., 2001). Recordings were referenced to the Cz electrode. The data were digitized with a 24-bit A/D converter at a 250 Hz sample rate.

### EEG data analysis

#### Data preprocessing

The continuous EEG data were digitally filtered between 0.1 and 30 Hz with zero-phase shift finite impulse response (FIR) filters. Trials with time-locked event responses were extracted from filtered data. The data were divided into four conditions: (1) partner acceptance, (2) partner rejection, (3) peer acceptance, and (4) peer rejection. The time period of a single trial was from 200 ms before event onset to 800 ms after event onset. Bad channels (defined as those with EEG max–min >200 μV after smoothing with a moving average of 80 ms long) were identified and replaced using spherical spline interpolation. Epochs with artifacts due to eye blinks or ocular movement were excluded. The remaining epochs for each participant were then averaged and baseline corrected to the first 200 ms period. The data were then re-referenced to the common average signal across all electrodes. In order to examine the latent nature of the N1, P3a and P3b component without the effects of overlapping ERP, the data were subjected to a two-step PCA/ICA component analysis (Luu et al., 2014; Lole et al., 2015).

#### Principal component analysis/independent component analysis

The average ERP data from each subject were entered into a spatiotemporal component analysis using the ERP PCA Toolkit version 2.50 (Dien, 2010, 2012). In this two-step component analysis, a temporal PCA decomposition using promax rotation was first conducted on the data with time point as a variable. The source of variance was accounted over subjects, conditions, and channels. The number of factors retained for temporal PCA decomposition can be guided by the parallel test (Ledesma and Valero-Mora, 2007). Following the temporal PCA, the spatial ICA decomposition was performed. ICA with informax method (Delorme and Makeig, 2004) used the channels as variables and the number of factors retained for the independent component can also be obtained by the parallel test. After this two-step component analysis, many spatiotemporal components were generated (number of temporal components multiplied by spatial components). The components to be considered for further analysis begun with elimination of all components that did not account for at least 0.5% of total variance (Dien, 2012). This removes the majority of junk factors that reveal noise. Next, we explored components of interest based on dipole-like scalp maps, spectral peak at typical frequency range, and identifiable as ERP components (Delorme et al., 2012). Once the spatiotemporal components were identified, repeated measure analysis of variance (ANOVA) without correction for multiple comparisons was tested for statistical significance (Dien, 2010, 2012) and the substantive significance (effect size) was also reported (Sullivan and Feinn, 2012). In this study, *P* < 0.05 was considered as significant.

#### Head model construction

The electrical head modeling (forward model) was created to include accurate brain tissue segmentation, 128-channel sensor position registration, and specification of conductivity values for each tissue. In the present study, an atlas head model was constructed from whole head MRI and CT images using BrainK software (Li et al., 2016). BrainK included five major steps: (1) Tissue segmentation: The MRI and CT images were co-registered prior to segmentation. Tissue segmentation classified and identified each image voxel into the following tissue types: air, eyeball, scalp, skull, cerebral-spinal fluid (CSF), and brain (gray and white matter). Scalp and skull were identified by CT image and CSF and brain were identified from MRI data. (2) Registration of sensor position: 128-channel sensor positions were registered to the respective scalp surface on the atlas head model. (3) Cortical surface reconstruction. (4) Dipole tessellation. (5) Talairach transformation: The MRI and CT images were then aligned with cortex volume from the MNI305 atlas with Talairach registration (Li et al., 2016). The locations of the dipoles were derived based on the method (Pascual-Marqui et al., 1994) by discretizing the gray matter volume of the MNI305 atlas. The tissue volumes were parceled into 7 mm voxels to form the computational elements; each voxel served as a source dipole location with three orthogonal orientation vectors (triples). This resulted in 1,732 dipole locations and 5,196 dipoles for our atlas head model.

For the complete head model, a lead field matrix (LFM), which describes the projection of current from each dipole source position to each EEG sensor position, was computed using the finite difference method (FDM; Salman et al., 2015). The following conductivity values (in Siemens/meter) assigned to each tissue type are based on previously reported literature values: Eyeball = 1.5, Scalp = 0.44, Skull = 0.018, CSF = 1.8, Brain = 0.25 (Dannhauer et al., 2012; Salman et al., 2015).

#### Cortical source estimation

Estimation of the cortical source was performed using GeoSource 3.0 software (EGI, Eugene, OR, USA). To estimate the activity on the cortex, source localization was performed with a constrained inverse model on each component using the Low Resolution Tomography (LORETA) method (Pascual-Marqui et al., 1994, 1999). The relationship between scalp and source can be stated as generalized linear models:

(1) $$\begin{array}{r} \Phi \left(t\right)=KJ\left(t\right)+\varepsilon \left(t\right), \end{array}$$

where Φ(*t*) is the EEG scalp potential measured at *N*_*e*_ = 128 electrodes, K is the lead field matrix, $J\in R^{N_{v}}$ are the activities of the source dipoles at the cortical surface, and ε(*t*) is generalized noise with covariance matrix *C*_ε_. Since inverse problem is ill-posed (*N*_*e*_ << *N*_*v*_), mathematical and physical constraints are added to obtain unique solution. Many linear inverse methods can be obtained as the solution of the minimization problem:

(2) Ĵ=arg min J{||Φ-KJ||2+λJTWJ},

(3) $$\begin{array}{r} Ĵ=K^{T}{\left[K^{T}K+\lambda W\right]}^{-1}\Phi , \end{array}$$

where ||Φ − *KJ*||^2^ is the data fidelity term, and λ is the regularization parameter which controls the influence of the constrains relative to minimizing the residual of the fit. *J* is the vector of source amplitudes (as defined above) and *W* defines the inverse technique (e.g., Minimum Norm, LORETA, sLORETA etc.). The LORETA method (Pascual-Marqui et al., 1994, 1999) employs regularization based on the 3D discrete Laplacian operator and LFM normalization. For LORETA inverse technique, *W* = *E*^*T*^*D*^*T*^*DE* where *D* is the Laplacian operator and *E* is a diagonal matrix corresponding to the LFM normalization. *E*=Ẽ ⊗ *I*_3_ is constant over three orthogonal orientation vectors to each source dipole and *D* =$\tilde{D}$ ⊗ *I*_3_ is the matrix for the Laplacian operator using a 7-point stencil (Hammond et al., 2013).

## Results

### dEEG data

Focusing on the results from the participant selection blocks, we analyzed the number of partner acceptance trials, meaning the number of times a participant would choose their romantic partner. Overall, participants chose their partner over an unfamiliar peer on average 65% of the time, indicating participants accepted their romantic partner more often than they rejected them.

**Figure 2:**
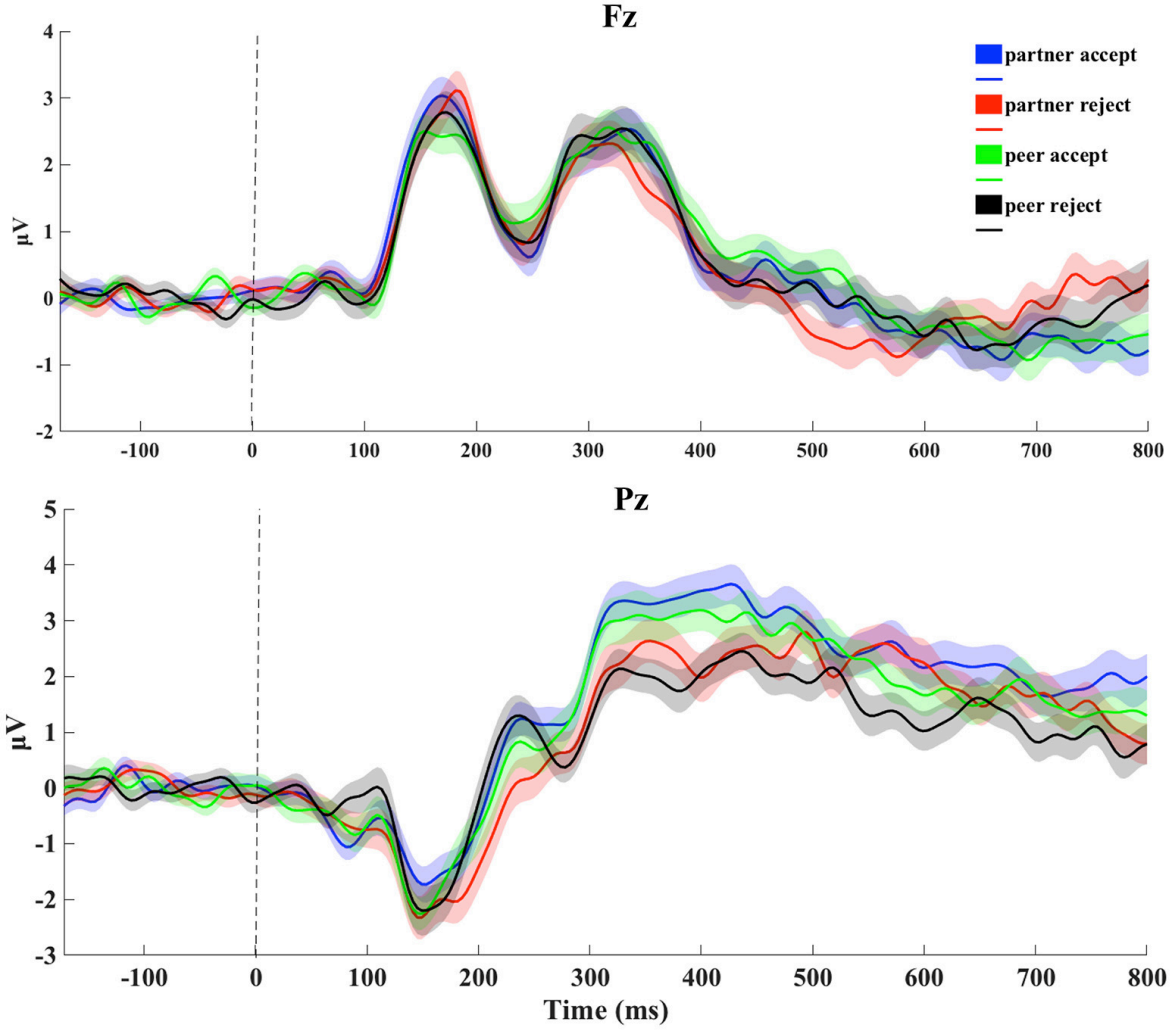
Original grand-average ERP waveforms at Fz and Pz. Signals were averaged across all subjects for four conditions (1,000 ms epoch). Stimulus onset was plotted as vertical dashed line. The thickness of the traces reflects the standard error through from subject-by-subject variations.

The grand average ERP data at Fz and Pz electrode sites from the feedback blocks for all four conditions is depicted in Figure 2. Upon visual inspection of the head surface (scalp) waveform, some typical ERP components, such as the N1 and P3, were clearly identifiable. At mediofrontal sites (channel Fz), no clear waveform difference was seen between conditions during 100–200 ms for the N1, whereas at centroparietal sites (channel Pz), the amplitude during 300–500 ms for both partner and peer acceptance conditions was higher than the partner and peer rejection conditions for the P3.

### Principal component analysis/independent component analysis

To decompose the overlapping ERPs, a two-step PCA/ICA was conducted. First, temporal PCA decomposed the individual ERP data into distinct temporal factors based on the parallel test. In this test, a scree plot derived from a fully random dataset was created and then compared with the scree plot obtained from the actual dataset. An intersection point of two datasets (random and actual) in the scree plot indicated the number of factors to be retained for the temporal decomposition. In our parallel test, 17 temporal factors, accounting for 91% of total variance, were retained. For the spatial ICA decomposition, eight discrete spatial factors were suggested to retain (76% of total variance) based on the parallel test. After this two-step component analysis, a total of 136 spatiotemporal components were generated. Based on the rules of component consideration described in PCA/ICA method section, four functionally distinct components were included for further component identification.

### Component 1

**Figure 3:**
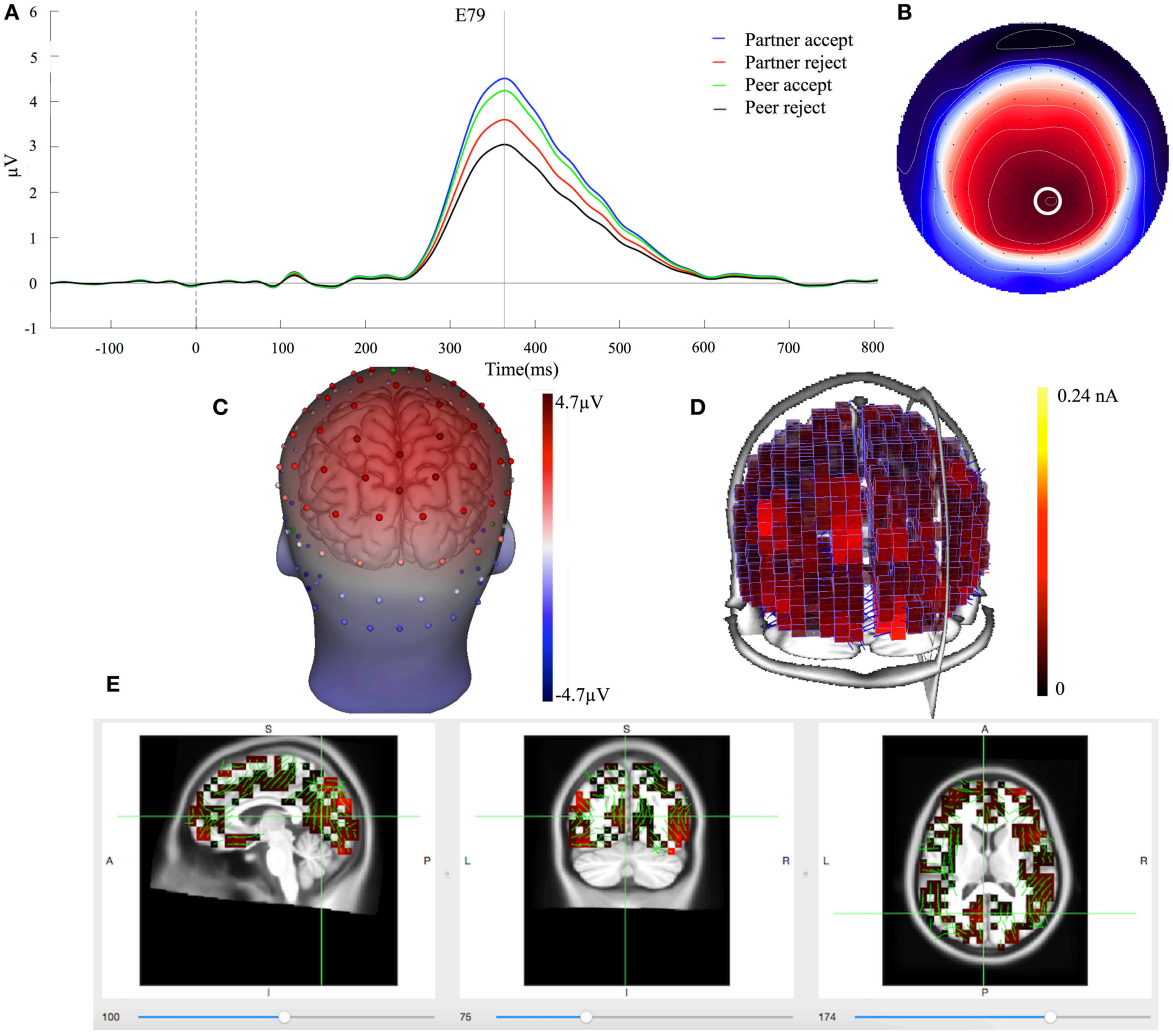
C1 component properties. **(A)** Component mean waveforms were at parietal sites at electrode E79 for all conditions. **(B)** Topography map of C1 was showing voltage distribution at 364 ms for partner acceptance condition. The white circle highlighted the location of channel E79 and block dots indicated all the 129 channels. Orientation was top looking down with nose at the top. **(C)** Scalp voltage distribution was on the atlas head model. **(D)** Source intensity on the vertical axis was shown in standardized units for component C1. **(E)** Source data was displayed and projected onto MRI slices. The area within the green border represented the posterior cingulate cortex.

The waveforms of the first component (C1) are displayed for all four conditions at centroparietal sites (Figure 3A) at the location indicated by a white circle (Channel 79) in the topography map in Figure 3B. The topography map of C1 displayed the peak activity at 364 ms and had positive peak amplitudes over the parietal area on the 3D head voltage distribution image in Figure 3C. This component is similar to late positive component (LPC) or P3b related component (Dien et al., 2004) in its topography and time course in the post-stimulus interval. Statistical analysis was performed on mean amplitudes of C1 (344–384 ms; EGI channels 62, 78, and 79) over each condition and subject. One-way ANOVA analysis revealed significant effects between partner acceptance (*M* = 4.32 μV) and partner rejection (*M* = 3.40 μV) conditions, *F*_(1, 74)_ = 3.99, *P* < 0.05, effect size = 0.32, and between peer acceptance (*M* = 4.06 μV) and peer rejection (*M* = 2.93 μV) conditions, *F*_(1, 74)_ = 6.82, *P* < 0.01, effect size = 0.42, resulting in larger amplitudes for both partner and peer acceptance conditions.

Cortical source estimation was performed using the LORETA constraint and a regulation constant of 10^−3^ on the averaged component waveform of all 75 subjects. Sources of C1 were obtained for the time point at 364 ms and displayed primary activities in the bilateral inferior temporal lobe (BA20), the bilateral parietal lobe (BA39 and BA7) and the posterior cingulate cortex (BA23 and BA31), as seen in Figure 3D. The C1 component also included activity in the bilateral visual associated lobe (BA17) and the bilateral parahippocampal (BA36). The bilateral inferior temporal lobe was stronger at the right hemisphere. Figure 3E shows the C1 generation in the PCC area on the MRI slices.

### Component 2

**Figure 4:**
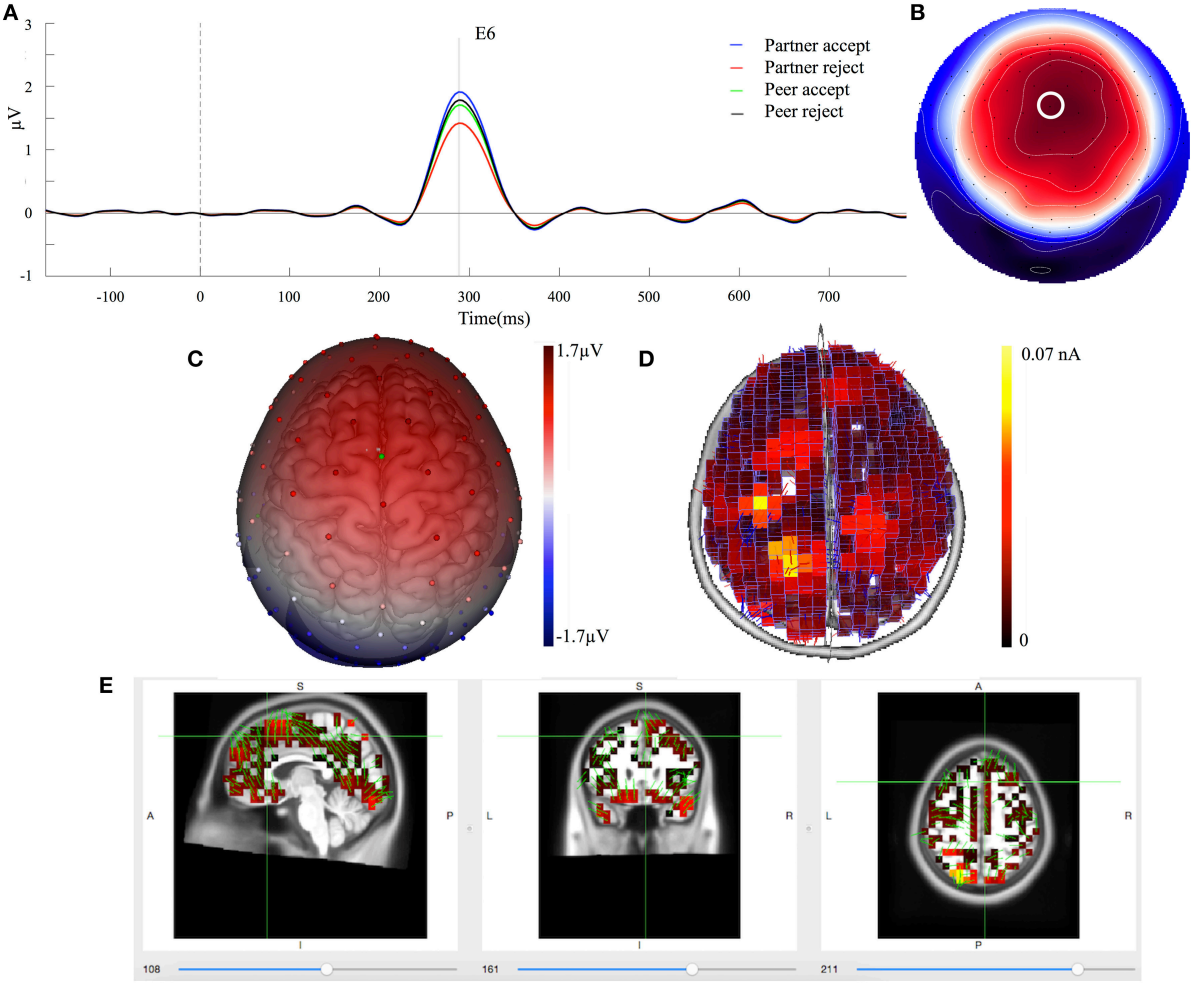
C2 component properties. **(A)** Component mean waveforms were at midfrontal sites at electrode FCz (E6) for all conditions. **(B)** Topography map of C2 was showing voltage distribution at 288 ms for partner acceptance condition. The white circle highlighted the location of channel E6. **(C)** Scalp voltage distribution was on the atlas head model. **(D)** Source estimation of C2 using LORETA was showed on brain cortex. **(E)** Source data was displayed and projected onto MRI slices. The area within the green border represented the anterior cingulate cortex.

The mean component waveforms and scalp topography of C2 are illustrated in Figures 4A,B. Mean waveforms are displayed at mediofrontal sites indicated by a white circle in the topography map for all conditions. This component had a positive deflection in the mediofrontal and a negative deflection in the occipital lobe shown in Figure 4C. It was most prominent at approximately 288 ms post-stimulus and resembled a P3a related component (Dien et al., 2004). The mean amplitudes for C2 (268–308 ms; EGI channels 6, 7, and 106) were extracted over each condition and subject. One-way ANOVA demonstrated that the waveforms differed significantly between partner acceptance (*M* = 2.05 μV) and partner rejection (*M* = 1.37 μV) conditions, *F*_(1, 74)_ = 4.18, *P* < 0.05, effect size = 0.33. No significant effects were found between peer acceptance (*M* = 1.71 μV) and peer rejection (*M* = 1.80 μV) conditions, *F*_(1, 74)_ = 0.11, *P* < 0.89, effect size = 0.05.

Source estimation of C2 showed primary activations in the bilateral parietal lobe (BA39 and BA7), the bilateral frontal lobe (BA8 and BA6) and the anterior cingulate cortex (BA32), as seen in Figure 4D. The bilateral inferior temporal lobe (BA20) and the bilateral occipital lobe (BA19) were also activated in the C2 component. Additionally, Figure 4E depicts the C2 generation in the ACC area on the MRI slices.

### Component 3 and 4

**Figure 5:**
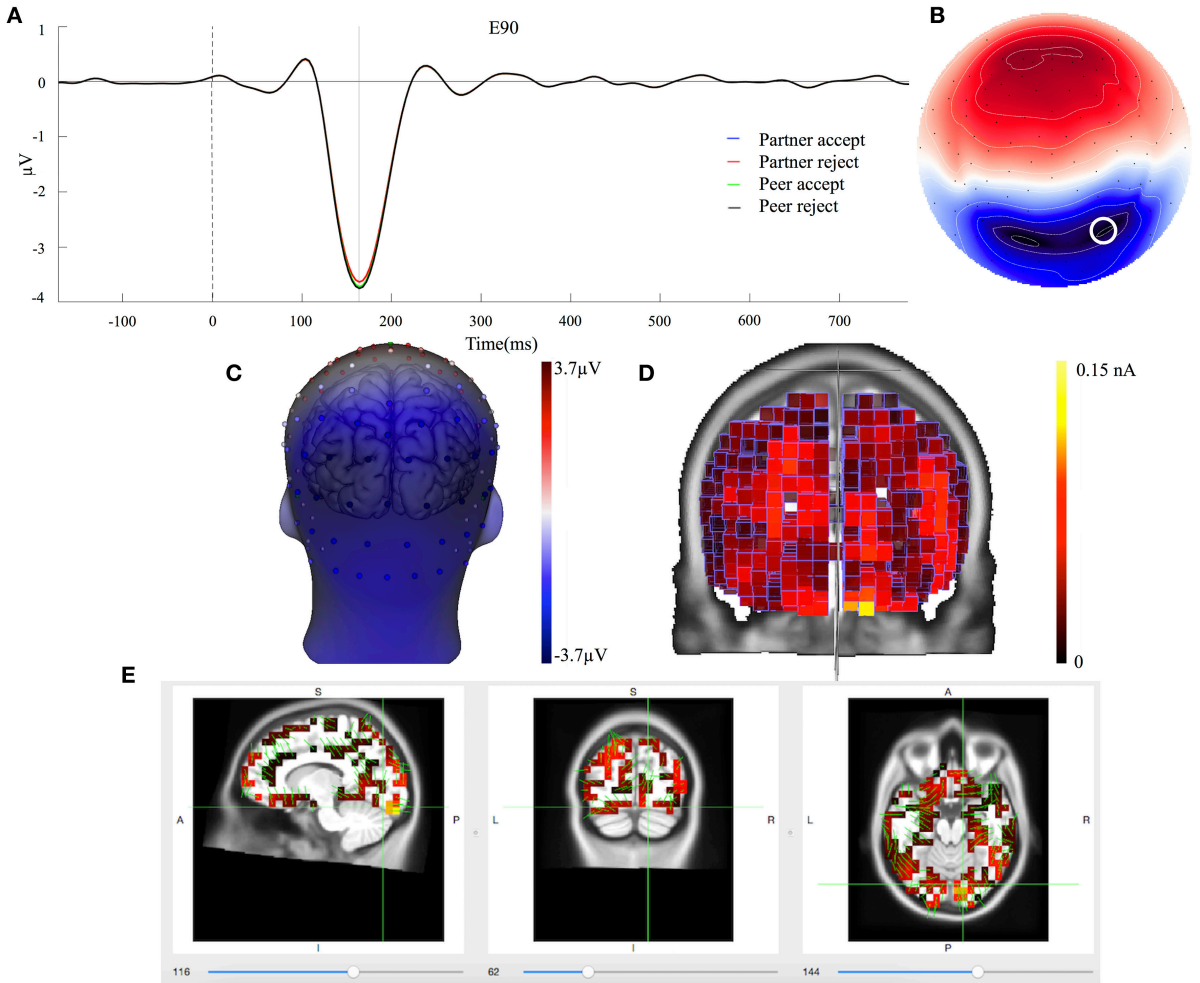
C3 component properties. **(A)** Component mean waveforms were at occipital sites at electrode E90 for all conditions. **(B)** Topography map of C3 was showing voltage distribution at 164 ms for partner acceptance condition. **(C)** Scalp voltage distribution was on the atlas head model. **(D)** Source estimation of C3 was showed on the brain cortex. **(E)** Source data was displayed and projected onto MRI slices. The area within the green border represented the visual cortex.

The C3 component was prominent at 164 ms, displayed in Figure 5A. The topography map had a positive deflection over the frontal lobe and a negative deflection in the occipital lobe, shown in Figures 5B,C. A typical N1 component is observed by its topography and time course in the post-stimulus interval (Poolman et al., 2008). The mean amplitudes of C3 (144–184 ms; EGI channels 70, 75, and 83) were extracted over each condition and subject for the statistical analysis. No significant effects were seen between partner acceptance (*M* = −3.34 μV) and partner rejection (*M* = −3.24 μV) conditions, *F*_(1, 74)_ = 0.05, *P* < 0.82, effect size = 0.04. Additionally, no significant differences were shown when comparing peer acceptance (*M* = −3.32 μV) and peer rejection (*M* = −3.35 μV) conditions, *F*_(1, 74)_ = 0.01, *P* < 0.94, effect size = 0.01. Source results displayed activities in the bilateral visual associated and primary visual lobes (BA18 and 17), the bilateral occipital lobe (BA19) and the posterior cingulate cortex (BA31 and BA23), seen in Figure 5D. The C3 component also included activities in the right fusiform area (BA37) and the bilateral inferior temporal lobe (BA20). The visual lobe activity was bilateral but stronger at the right hemisphere. Lastly, Figure 5E depicts the C3 generation in the visual cortex area on the MRI slices.

**Figure 6:**
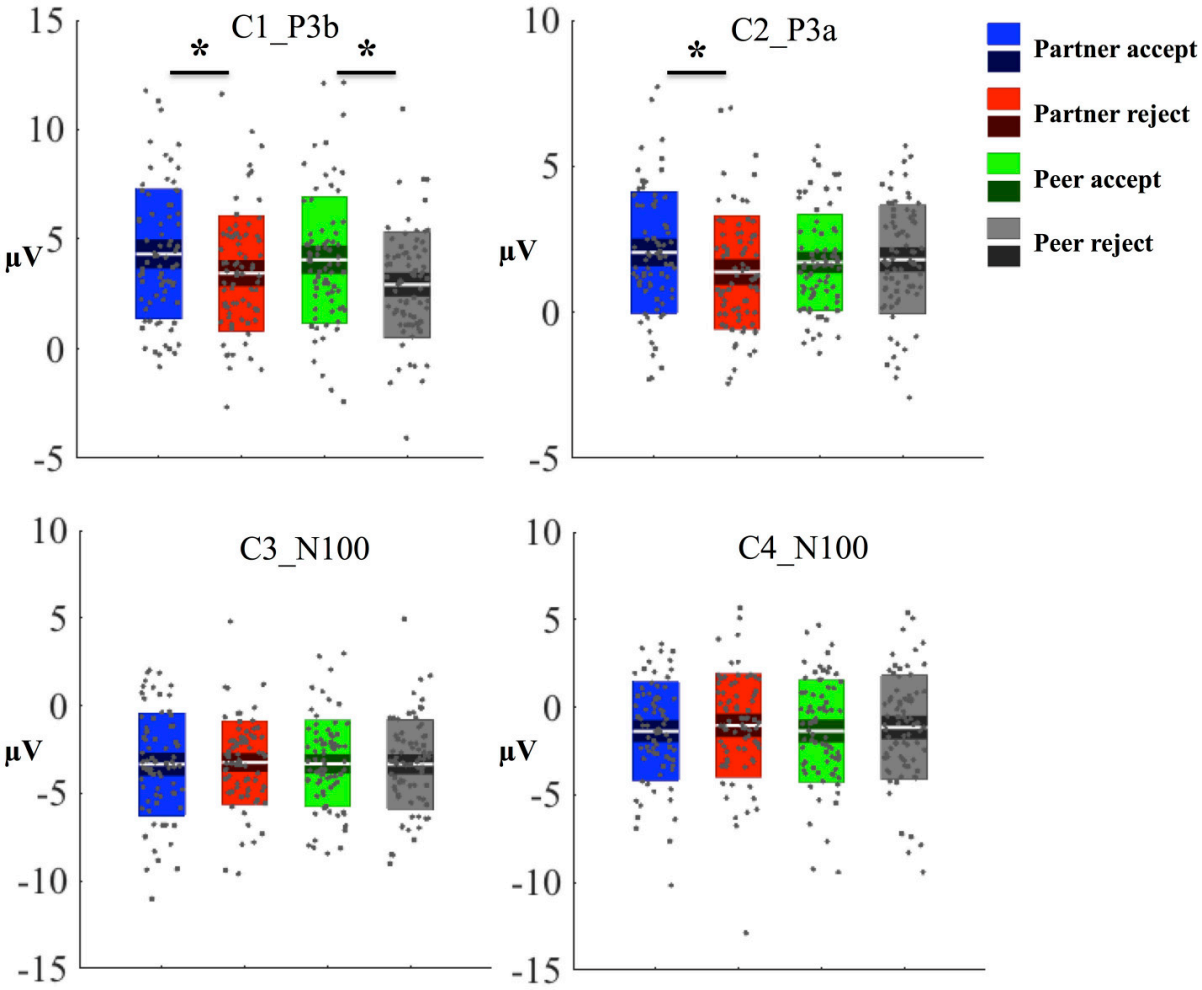
Experimental effects for each component and condition. Amplitude from each subject and condition was plotted as a gray dot and the mean amplitude was indicated as a whit line. The standard error (SEM, 95% confidence interval) and standard deviation (SD) were sub-plotted with white lines as boxes. One-way ANOVA was used to test statistical significant (^*^P < 0.05) between conditions.

The properties of the C4 component are similar to the C3 component based on its topography map (similar to Figure 5B) and time information (C4 prominent at 124 ms). The mean amplitudes of C4 (104–144 ms; EGI channels 70, 75, and 83) were extracted over each condition and subject. No significant differences were found between partner acceptance (*M* = −1.37 μV) and partner rejection (*M* = −1.04 μV) conditions, *F* (1, 74) = 0.5, *P* < 0.48, effect size = 0.12. No change was obtained between peer acceptance (*M* = −1.36 μV) and peer rejection (*M* = −1.15 μV) conditions, *F*_(1, 74)_ = 0.19, *P* < 0.67, effect size = 0.07. Experimental effects for each component and condition are displayed in Figure 6. Source results indicated activations were in the bilateral visual associated and primary visual lobe (BA18 and BA17), the bilateral occipital lobe (BA19), the bilateral parietal lobe (BA39 and BA7), and the PCC (BA31 and BA23).

## Discussion

### C1 and C2 components

After the two-step PCA/ICA analysis, C1 and C2 are statically separated from overlapping activity, making their quantification more accurate and improving the statistical analysis. C1 has positive peak amplitude at 364 ms post-stimulus over the parietal area on the head surface (Figure 3B), while component C2 peaks at 288 ms post-stimulus and elicits a stronger frontocentral positive going wave (Figure 4B). The patterns of these results indicate that C1 and C2 can be identified respectively, as P3b and P3a related components with different scalp distributions and underlying cortical networks. The P3a has more of an anterior distribution compared to the P3b. A number of findings suggest that the P3a reflects a stimulus driven attention shift or social reward, while the P3b reflects task relevant information processing (Polich, 2007). P3a may be generated from frontal if sufficient attention is involved with task stimuli and P3b appears when information updating activations promote memory operations in the temporal-parietal junction brain area (Brazdil et al., 2001, 2003).

As the adolescents evaluated the response of romantic partners and unfamiliar peers in the Chatroom Interact Task, the initial electrophysiological response (P3a), maximal over frontomedial regions, was enhanced for acceptance (vs. rejection) responses of partners, but not for responses of peers. The implication may be that with strong expectations for the partner's evaluation the initial orienting of attention indexed by the P3a was differentially modulated by the partner's feedback. In contrast, the somewhat later (364 ms) P3b component showed a greater response for acceptance than rejection for both the partner and peer. This suggests that the more extended cognitive evaluation indexed by the P3b is adequately engaged in interpreting the acceptance feedback even from peers. Higher P3a and P3b amplitudes to acceptance are an extension to previous studies (Gunther Moor et al., 2010; van der Veen et al., 2014), implicating initial attention is paid to partner acceptance, whereas later processing is more oriented toward general social acceptance. Overall, these findings support the notion that adolescents' desires for affiliation are high, which are particularly focused on partner acceptance.

In the traditional ERP literature (Polich, 2007), the differentiation between P3a and P3b is made on the basis of their differential latency. However, because they are overlapping, statistical characterization of these components is improved through the statistical decomposition from methods like PCA and ICA. In the present research, the two-step temporal PCA then spatial ICA analysis provided superior separation of these components than either component analysis alone, adding further evidence to the robustness of this analytic method (Dien, 2010, 2012).

### Comparison of cortical activation of C1/C2 components with fMRI

Source estimation of the ERP components can be improved by whole head spatial sampling (including sensors over both the superior and inferior head surface) and a high resolution electrical conductivity head model (Song et al., 2015). In addition to its more frontal head surface distribution, source analysis suggests the P3a is generated in frontal, parietal, and anterior cingulate areas in the control of attention. Source analysis of the P3b suggested it is localized in parietal, temporal, and posterior cingulate areas that are engaged in information evaluation. Together, P3a and P3b reveal a widely distributed network pathway engaging both frontal and temporal-parietal areas (Polich, 2003). These source patterns associated with P3a and P3b generation are in line with other EEG studies (Volpe et al., 2007; Li et al., 2015; Lole et al., 2015), which have been carried out with different experimental designs and paradigms. Particularly, these sources are consistent with other reports (Mulert et al., 2004a,b; Volpe et al., 2007) that used the LORETA inverse solution. Furthermore, our source findings are also confirmed by the patterns of fMRI hemodynamic responses in the frontal, temporal, and parietal activations for distractor stimulus (P3a) and target stimulus (P3b) processing (Bledowski et al., 2004a,b).

### C3 and C4 components

In the present study, two more spatiotemporal components were extracted. C3 peaks at 164 ms post-stimulus and displays a positive deflection in the frontal lobe and a negative deflection in the occipital lobe (Figure 5B). Additionally, C4 peaks at 124 ms post-stimulus and presents a similar scalp distribution as C3. The main difference between C3 and C4 is their temporal course (164 vs. 124 ms), which is separated by PCA decomposition. The C3 and C4 can be considered as typical N1 related components, based on their topography maps and time course (Mangun et al., 1994; Senholzi and Ito, 2012). The apparent network patterns of the C3 and C4, including the visual, occipital and PCC areas, are consistent with the major locations of the N1 (Im et al., 2007; Poolman et al., 2008). Interestingly, the differing cortical networks for these similar visual components appear to reflect sources in right fusiform for C3 and bilateral parietal lobe for C4. N1 is elicited by the Chatroom Interact Task in response to visual stimulus change. Nonetheless, no significant differences were observed between partner and unfamiliar peer groups, nor between acceptance and rejection conditions. The absence of condition differences implies that adolescents do not track differences in feedback during this early stage of visual processing.

### Limitations and future research

Although source analysis provided some suggestions of widespread frontal and posterior networks engaged in attentional orienting and cognitive evaluation, further understanding of the specific anatomy of these networks would require closer comparisons with imaging methods such as fMRI. Furthermore, the averaging of multiple trials in the ERP methodology may limit the understanding of time dynamics of individual trials, such as oscillatory EEG changes. In contrast to conventional ERP and PCA/ICA component analyses, EEG spectral analyses have revealed that EEG oscillations in particular frequency bands are functionally related to cognitive processing and behavior (Pfurtscheller and Lopes da Silva, 1999; Kuo et al., 2014). For example, our motor study has shown that the most robust effects were the beta-band (14–30 Hz) event-related desynchronizations (ERD, power decreases), appearing in all individuals, consistently localized to the hand region of the primary motor cortex, and consistently aligned with fMRI localizations (Kuo et al., 2014). In another study, the task-induced alpha oscillation has specific functional rules where low alpha ERD (8–10 Hz) is linked to attention and high alpha ERD (10–12 Hz) is associated with memory (Klimesch et al., 2003; Schack et al., 2005). Further research using spectral analysis may be useful for determining oscillatory dynamics in the process of understanding social feedback. In addition, the functional connectivity network analysis examines the patterns of coherence or correlations among cerebral networks, which is another approach integrated to EEG-based neuroimaging techniques. In the present study, the temporal-spatial analysis of the ERP separated the major cortical networks, but did not characterize their dynamic interaction. In future research, understanding the connection, communication, and causality among these source areas will be important for understanding social feedback, considering the brain-to-brain resonance in both partner and peer communications (Dumas et al., 2012; Yun et al., 2012; Hassan et al., 2015; Chennu et al., 2016).

## Conclusion

The present study suggests that PCA/ICA component analysis can separate functionally distinct components effectively with different time courses and underlying cortical networks that contribute to the perceived social feedback from romantic partners and unfamiliar peers. Adolescents exhibited more attention to partner acceptance early in their processing of the neural communication (P3a) and similar cognitive processing for both partner and peer acceptance in the next stage (P3b) of cognitive evaluation. Our study provides a potentially influential methodological foundation for the development of future research, clinical treatment, and interventions.

## Author contributions

CCK, TH, and TD contributed to the design of the study. TH and AE were responsible for data acquisition. CCK and DT contributed to data analysis. All authors contributed to the interpretation and preparation of the manuscript. All authors approve the version that is currently under consideration and acknowledge that they are accountable for all aspects the work.

### Conflict of interest statement

The authors declare that the research was conducted in the absence of any commercial or financial relationships that could be construed as a potential conflict of interest.
